# Maternal Vitamin C Deficiency during Pregnancy Persistently Impairs Hippocampal Neurogenesis in Offspring of Guinea Pigs

**DOI:** 10.1371/journal.pone.0048488

**Published:** 2012-10-31

**Authors:** Pernille Tveden-Nyborg, Lucile Vogt, Janne G. Schjoldager, Natalie Jeannet, Stine Hasselholt, Maya D. Paidi, Stephan Christen, Jens Lykkesfeldt

**Affiliations:** 1 Department of Veterinary Disease Biology, Faculty of Health and Medical Sciences, University of Copenhagen, Copenhagen, Denmark; 2 Institute of Infectious Diseases, University of Bern, Bern, Switzerland; 3 Department of Neurology, University of California San Francisco, San Francisco, California, United States of America; Hôpital Robert Debré, France

## Abstract

While having the highest vitamin C (VitC) concentrations in the body, specific functions of VitC in the brain have only recently been acknowledged. We have shown that postnatal VitC deficiency in guinea pigs causes impairment of hippocampal memory function and leads to 30% less neurons. This study investigates how prenatal VitC deficiency affects postnatal hippocampal development and if any such effect can be reversed by postnatal VitC repletion. Eighty pregnant Dunkin Hartley guinea pig dams were randomized into weight stratified groups receiving High (900 mg) or Low (100 mg) VitC per kg diet. Newborn pups (n = 157) were randomized into a total of four postnatal feeding regimens: High/High (Control); High/Low (Depleted), Low/Low (Deficient); and Low/High (Repleted). Proliferation and migration of newborn cells in the dentate gyrus was assessed by BrdU labeling and hippocampal volumes were determined by stereology. Prenatal VitC deficiency resulted in a significant reduction in postnatal hippocampal volume (P<0.001) which was not reversed by postnatal repletion. There was no difference in postnatal cellular proliferation and survival rates in the hippocampus between dietary groups, however, migration of newborn cells into the granular layer of the hippocampus dentate gyrus was significantly reduced in prenatally deficient animals (P<0.01). We conclude that a prenatal VitC deficiency in guinea pigs leads to persistent impairment of postnatal hippocampal development which is not alleviated by postnatal repletion. Our findings place attention on a yet unrecognized consequence of marginal VitC deficiency during pregnancy.

## Introduction

Vitamin C (VitC) deficiency can have detrimental effects in the brain of juvenile guinea pigs. We have previously shown that a postnatal VitC deficiency in guinea pigs results in an impaired spatial memory function and a significant reduction in the number of neurons in the cornu ammonis (CA)1, the CA2–3 and the dentate gyrus (DG) of the hippocampus, unveiling a histomorphometric link between VitC deficiency and a negative impact on brain function [1]. During development, the brain is particularly vulnerable to low levels of antioxidants because of a high growth rate resulting in increased cellular metabolism and an immature antioxidant network leading to redox imbalance [2]. Thus, levels of VitC are increased in several tissues, including the brain during early life [3], and a reduced level has been shown to increase markers of oxidative stress [2], [4].

The brain undergoes multiple steps of cellular proliferation, differentiation and maturation during development. *In vitro* data have shown that VitC plays a key role in the regulation of neuronal differentiation, maturation and neurite formation [5]–[7], implying that a negative impact of VitC deficiency on brain development could be expected. This has also been supported by *in vivo* findings establishing that VitC transport to the brain is essential for perinatal survival [8], [9]. Within the brain, the hippocampus has one of the highest VitC concentrations [10]–[12] and a corresponding high level of the VitC transporter–the *sodium dependent vitamin C co-transporter 2* (SVCT2) [13], [14].

Like humans, guinea pigs are unable to synthesize VitC due to a mutation in the gene encoding for L-gulonolactone oxidase, making guinea pigs a highly valuable *in vivo* model for VitC deficiency. The guinea pig is a precocial species with the peak of overall cellular growth in the brain (‘the brain growth spurt’) occurring before birth [15], [16]. Based on our previously shown VitC deficiency induced reduction in hippocampal neurons and impairment of Morris Water Maze (MWM) performance in young guinea pigs, we therefore hypothesized that these effects may also occur when deficiency is imposed during gestation, a condition that can be observed in large subpopulations of pregnant women.

The aim of the current study was to investigate the effect of prenatal VitC deficiency on postnatal hippocampal development and function in guinea pigs and whether any such effect could be reversed by VitC repletion immediately after birth. Hippocampal development was assessed by determining the rate of proliferation of newborn cells in the DG at postnatal day (P)10, their survival at P27, and calculating hippocampal volumes on P10, P27 and P70. We found that prenatal deficiency negatively affects the postnatal hippocampal volume and cellular migration within the hippocampus. These findings were persistent despite re-introduction of high VitC levels immediately after birth.

## Materials and Methods

### Ethics

The study was approved by the Danish Animal Experimentation Inspectorate under the Ministry of Justice (Permit number: 2007/561-1298). Surgery prior to euthanasia was performed under Isoflurane anesthesia and all efforts were made to minimize suffering.

### *In Vivo* Study

**Figure 1:**
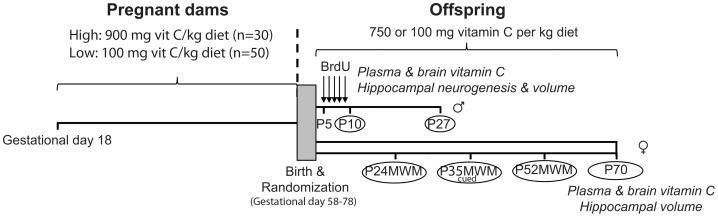
Overview of *In Vivo* Study. Eighty pregnant guinea pigs were weight-stratified into two groups at gestational day 18. Group High (n = 30) was assigned to a diet containing 900 mg of vitamin C per kg diet and group Low (n = 50) received a diet containing 100 mg of vitamin C per kg. Otherwise, diets were identical. Newborn pups were assigned to either of four dietary groups (CTRL, DEF, DEPL or REPL) by weight stratification. Weaned male pups (n = 83) received a daily BrdU injection (100 mg/kg body weight) between post-natal day (P) 5 and P9 and were sacrificed either at P10 or P27. Female animals (n = 77) did not receive BrdU. A subset (n = 36) were submitted to two consecutive tests in the non-cued Morris water maze (MWM) initiated at P24 and P52. A cued test (c-MWM) was performed on age-matched pups from both CTRL and DEF groups (n = 5 and n = 6, respectively) at P35. All female pups were sacrificed at approximately two months of age (P70). Biochemical and histochemical analyses were performed as indicated on the scheme.

Eighty Dunkin Hartley guinea pigs (Charles Rivers Lab, Kieslegg, Germany) at gestational day 18, e.g. within the first trimester (gestation length is 58–78 days [17]), were equipped with a subcutaneous (s.c.) microchip for identification, and randomized into weight stratified dietary groups receiving High (900 mg, n = 30) or Low (100 mg, n = 50) levels of VitC per kg diet (quality controlled diets; Special Diets Services, SDS, Witham, England). Upon arrival, maternal VitC levels in plasma were obtained by blood sampling (39.8±11.8 µM). The dose of 100 mg VitC/kg feed has been shown to result in a non-scorbutic VitC deficiency in guinea pigs even during long-term studies [18], [19]. After birth, newborn pups were randomized again to either High (750 mg) or Low (100 mg) VitC per kg diet, giving a total of four different postnatal dietary feeding regimens: High/High (control, CTRL); High/Low (postnatal depletion, DEPL; only male pups), Low/Low (pre/postnatal deficiency, DEF); and Low/High (postnatal repletion, REPL). See Figure 1 for graphical overview of study design.

Pups were weighed and micro-chipped (s.c. in the neck) within 24 hrs after birth. Of the total of 285 live born pups, 168 were included in the current study. There was no statistically significant difference in the number of live born pups between dietary groups or effects of dietary VitC or gender on birth weight of pups. Four dams proved not to have conceived and were subsequently euthanized; one dam was euthanized during gestation.

Male pups (n = 91) were randomly allocated to experimental groups stratified by birth weights. Within the first five days of life, four pups born from High and four pups from Low mothers died, leaving n = 83. Pups were weaned five days after birth, post-natal day (P)5, (except from DEPL, which were weaned at P2) and group-housed in approved guinea pig housing racks. Pups received BrdU intraperitoneally (100 mg/kg Sigma-Aldrich, St Louis, MO, USA) once daily from P5–P9, as described previously [20]. Pups were euthanized either on P10 or P27. During the study, six animals died or were euthanized. Autopsies revealed gingivitis and dental malformations in two pups, whereas no specific macroscopic findings were recorded for the remaining four pups.

Female pups (n = 77) were randomly allocated to the three dietary groups CTRL, DEF and REPL (n = 22 for each group) and weaned around P7. Twelve animals were allocated from each dietary group (stratified according to birth weights and age) to be tested in a non-cued MWM. Additional age-matched pups were included in the CTRL and DEF groups (n = 5 and n = 6 respectively) to be tested in a cued MWM. Pups were euthanized between P56–P79 referred to in the text as P70.

Unless stated otherwise, animals were housed in floor pens, allowed feed, dried hay and water ad libitum, and tended several times daily by trained staff. Body weights were recorded at least twice a week. At arrival and two times during gestation, 300 µL blood samples were taken from the v. saphena at its superficial course on tibia in eight random dams of each group for verification of VitC status (data not shown). Likewise, blood samples were taken from female pups around P35 and P63 (data not shown).

### Euthanasia

Animals were anesthetized by inhalation of Isoflurane (Isoba Vet 100%, Intervet International, Boxmeer, The Netherlands). Upon the disappearance of voluntary reflexes, thoracotomy was performed and an intracardial blood sample was collected by 5 mL syringe and 18G hypodermic needle previously flushed with 15% tripotassium-EDTA [21]. Euthanasia was achieved by exsanguination and subsequent decapitation. Blood samples were immediately centrifuged and stabilized after sampling. Tissue samples were removed and placed in ice-cold phosphate buffered saline (PBS) prior to freezing on dry ice and subsequent −80°C. The brain was excised, weighed and divided into left and right hemispheres (by section through the cerebral longitudinal fissure). The hemispheres were randomly assigned to either fixation (4% paraformaldehyde (PFA) in PBS, 0.15 M, pH 7.4 to be transferred to 1% PFA in PBS within 48 hrs) or storage at −80°C to be used for biochemical analyses.

### Morris Water Maze Test

All performances in the MWM were recorded by CCD-camera (Camcolmha6, Velleman, Gaverer, Belgium) in mpeg-2 format and later analyzed by Ethovision XT software ver. 6.0 (Noldus Information Technologies, Wageningen, The Netherlands). Swim patterns applied in the non-cued MWM were categorized as described previously [1], [22].

### Cued Morris Water Maze Test

To test for an effect of vitC deficiency on the overall non-spatial abilities to perform a water maze test [23], [24], a subset of female age-matched pups (CTRL (n = 5) and DEF (n = 6)) were subjected to a cued water maze on P35. Outside cues were hidden/removed and the platform was cued by a small flag (10×5 cm, 14 cm high [23]) and remained in the same quadrant during the three days of trial. Animals were exposed to two x four swims on trial day one and two, and four swims on trial day three [23]–[26]. Swim-protocol followed that of the non-cued water maze.

### Non-cued Morris Water Maze Test

Animals (n = 36) enrolled in the non-cued MWM [27] were subjected to two consecutive tests starting around P24 and P52, respectively. The test protocol consisted of a five day acquisition phase followed by a four day rest prior to the retention trial as described previously [1], [23]. The platform was placed consistently in the same quadrant during the acquisition phase and animals were submitted randomly to the trial. During the P24–test, two animals were excluded leaving n = 34 (CTRL: n = 12; DEF: n = 11; REPL: n = 11).

### Vitamin C Analysis

Ascorbate (ASC) and its oxidation ratio (the concentration of dehydroascorbic acid (DHA), i.e. the oxidized form of VitC, to that of total VitC in percent) were analyzed by HPLC with coulometric detection as described previously [28], [29].

### Hippocampal Volume and Proliferation/migration of Newborn Cells

6 µm thick paraffin-embedded coronal sections were cut on a microtome (HM335E, Germany), placed on Superfrost® Plus Microscope slides and dried on a heating plate for 24 hrs at 40°C. Five consecutive sections were taken for every region each 400 µm apart, spanning the entire depth of the hippocampus (∼10 regions per animal). Cutting of the coronal sections was done with the guidance of the guinea pig brain atlas by Rapisarda and Bacchelli [30].

Nissl staining was performed on the first section of every region after deparaffinization and rehydration. Stained sections were scanned with a Polaroid print scan 35plus and the hippocampal area was determined in a blinded fashion with ImageJ (Wayne Rasband, NIH). The hippocampal volume for each animal was then calculated by the Cavalieri principle, according to the formula [20]: V = ∑ areas * distance between sections.

To assess proliferation and migration of newborn cells in the hippocampus, immunofluorescence staining was performed as described previously [31] on four out of eight coronal sections covering the depth of the DG, making each region 800 µm apart. Deparaffinized and rehydrated sections were boiled for 15 min in 10 mM sodium citrate buffer, pH 6.0 for optimized antigen retrieval of BrdU, nuclear DNA and cell markers [32]. Sections were co-stained with polyclonal sheep anti-BrdU (Novus MB 500-235) and mouse monoclonal anti-NeuN (Chemicon MAB377) or mouse monoclonal anti-GFAP (Chemicon MAB360), and counterstained with 4′-6-diamidino-2-phenylindole (DAPI) to visualize cell nuclei. The BrdU-positive cells in each part of the DG (hilus, subgranular zone [SGZ] and granular layer [GL]) were counted in a blinded fashion on stitched images taken at 20X magnification. Images were acquired on a wide-field epifluorescence microscope (AxioImager M1) using AxioVision Digital Imaging software (Carl Zeiss MicroImaging GmbH, Jena, Germany).

### Data Analysis

Statistical analysis of variance was performed on the collected data for animal body weight, brain weight, VitC concentration in brain and plasma, MWM performance, hippocampal volume, and number of BrdU positive cells in the different regions of the DG (Statistica v 7, Statsoft, Tulsa, OH, USA). Two-way ANOVA was applied to the stereological, immunohistochemical and biochemical data. In case of significance, Tukey’s post hoc test was applied. For analysis of MWM data, repeated measures ANOVA was applied to the cued test and acquisition phase of the non-cued MWM. Retention trial of the non-cued MWM was analyzed by one-way ANOVA. Comparison of swim-patterns was done with Fishers exact test. A P-value <0.05 was considered to be statistically significant.

## Results

### Effect on Body Weight and Brain Weight

During the study, no animals developed clinical signs of scurvy in accordance with our expectations and previous validation of a diet of 100 mg VitC/kg feed to be non-scorbutic [1], [18], [19], [33]. Postnatally depleted (DEPL) pups exhibited a significant lower body weight than controls (CTRL) (P<0.001), whereas the remaining groups were not significantly different from CTRL (Table 1). VitC deficiency did not have an effect on brain weight, except specifically for postnatally repleted (REPL) animals at P10. As expected, brain and body weight increased with age (p<0.001; Table 1).

**Table 1:** Brain weight and histological data from the different time-points.

	*P10 (male)*				*P27 (male)*				*P70 (female)*			*Two-way ANOVA*	
	CTRL(n = 10)	DEF(n = 10)	DEPL(n = 11)	REPL(n = 10)	CTRL(n = 10)	DEF(n = 9)	DEPL(n = 7)	REPL(n = 10)	CTRL(n = 10)	DEF(n = 10)	REPL(n = 10)	Effect of dietary regimen	Effect of age
Body weight (g)	115.3±14.6	124.2±14.4	105.4±23.5	103.4±19.5	236.9±38.3	244.9±30.2	159.3±48.7**	223.4±49.0	438.6±68.9	460.8±40.7	482.1±96.1	p<0.001DEPL < CTRL***	p<0.001P27>P10***P70>P10***P70>P27***
Brain weight (g)	2.80±0.13	2.77±0.14	2.66±0.18	2.58±0.18*	3.15±0.26	3.06±0.14	3.07±0.32	3.10±0.24	3.63±0.22	3.46±0.19	3.51±0.26	ns	p<0.001P27>P10***P70>P10***P70>P27***
Hippocampal volume (mm^3^)	55.7±3.7*(n = 9)*	51.6±2.3*(n = 9)*	56.5±5.1*(n = 9)*	47.2±5.8**	59.2±6.2 *(n = 9)*	54.1±5.2	62.3±7.1	53.5±3.7	62.7±7.9	54.4±6.7	55.7±4.0*(n = 8)*	p<0.001DEF < CTRL***REPL < CTRL***	p<0.001P27>P10**P70>P10***

Data is presented as mean±SD and were analyzed by two-way ANOVA using postnatal age and dietary regimen as factors followed by Tukey’s test for posthoc comparisons in case of statistical significance.

* p<0.05;

** p<0.01;

*** p<0.001 compared to CTRL animals. ‘ns’: not significant. Within each time point, one-way ANOVA was used.

### Biochemical Analysis

Plasma VitC concentration reflected the applied dietary regimes (Table 2), the deficient guinea pigs (DEF and DEPL) being consistently significantly lower in plasma VitC than CTRL counterparts (p<0.001). Plasma VitC concentrations in REPL were also significantly lower than CTRLs (P<0.01 by ANOVA), particularly manifested at P27 (P<0.001). A similar pattern was observed for total ASC concentrations in the brain (DEF and DEPL vs CTRL, P<0.001), except that brain ASC was not lower in REPL, demonstrating the preferential repletion of the brain. Plasma ASC concentrations increased with age (p<0.05), while brain ASC concentration (p<0.001) and DHA declined significantly with age (p<0.01). At P70, MWM animals displayed significantly lower levels of ASC in plasma, but not brain, compared to non-mazed animals (P<0.05). ASC oxidation ratio was assessed in the brain as a measure of redox imbalance [34], [35]. DEF animals showed significantly higher ASC oxidation ratio compared to the other groups (P<0.001; Table 2). MWM animals showed a several-fold higher ASC oxidation ratio compared to non-mazed animals (P<0.001).

**Table 2:** Vitamin C status in guinea pigs at the different postnatal time points.

	*P10 (male)*				*P27 (male)*				*P70 (female)*			*Two-way ANOVA*		*P70 (female, MWM)*			*Effect of MWM*
	CTRL(n = 10)	DEF(n = 10)	DEPL(n = 11)	REPL(n = 10)	CTRL(n = 10)	DEF(n = 9)	DEPL(n = 7)	REPL(n = 10)	CTRL(n = 10)	DEF(n = 10)	REPL(n = 10)	Effect ofdietaryregimen	Effectof age	CTRL(n = 12)	DEF(n = 11)	REPL(n = 11)	
Plasma total ascorbate (µM)	31.3±9.7	3.3±2.9***	5.7±6.3***	24.6±13.0	54.8±20.1	3.5±1.4***	2.6±1.5***	24.1±11.8***	51.8±22.4	4.0±3.2***	57.3±13.6	p<0.001DEF < CTRL***DEPL < CTRL***REPL < CTRL**	p<0.05P10<P27**P10<P70***P27<P70*	43.9±21.8	5.6±4.2***	35.5±15.8	P<0.05
Brain totalascorbate(µmol/g tissue)	1.67±0.10	0.76±0.20***	1.42±0.13**	1.80±0.14	1.58±0.13	0.63±0.20***	0.83±0.38***	1.54±0.09	1.34±0.15	0.45±0.25***	1.45±0.04	p<0.001DEF < CTRL***DEPL < CTRL***	p<0.001P10>P27**P10>P70***P27>P70**	1.50±0.13	0.59±0.34***	1.56±0.12	ns
Brain ascorbate oxidation ratio(% DHA of total VitC)	3.7±2.5	20.0±10.1***	8.4±6.7	5.8±2.9	4.3±9.4	6.7±3.3	4.5±6.7	3.8±3.5	4.5±2.8	9.5±4.6**	3.1±2.2	p<0.001DEF > CTRL***	p<0.01P10>P27**P10>P70*	21.8±3.7	31.1±4.7***	24.5±5.4	P<0.001

Data is presented as mean±SD and were analyzed by two-way ANOVA using postnatal age and dietary regimen as factors followed by Tukey’s test for posthoc comparisons in case of statistical significance.

* p<0.05;

** p<0.01;

*** p<0.001 compared to CTRL animals. ns: not significant. Within each time point, one-way ANOVA was used. Effect of the MWM trial on biochemical markers were tested by two-way ANOVA using MWM and dietary regimen as factors.

### Effect on the Hippocampus

Animals subjected to prenatal VitC deficiency (DEF and REPL) displayed a significant decrease in mean hippocampal volume (Table 1). Hippocampal volumes were about 10 to 15% lower in the prenatally-deficient animals (DEF and REPL) compared to the corresponding counterparts (P<0.001 by 2-way ANOVA). In contrast, no decrease in hippocampal volume was observed in DEPL animals at the two time points investigated. Hippocampal volume increased with age (p<0.001), although no significant increase was observed from P27 to P70, suggesting that the hippocampus does not grow any further after this time point.

**Figure 2:**
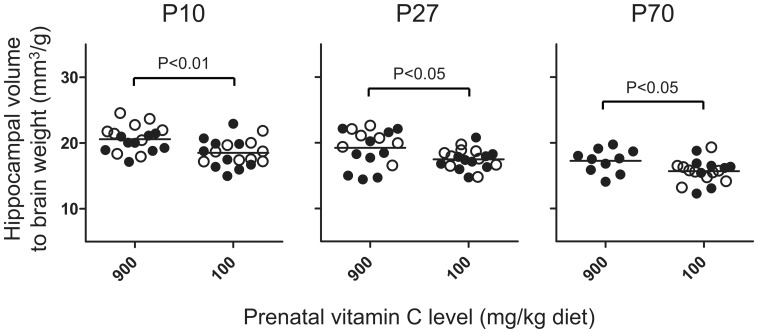
Persistently lower hippocampal volume corrected for brain weight in prenatally vitamin C-depleted animals. Results are presented as the grand mean for each prenatal treatment (i.e., either low or high). Closed circles (•) represent high and open circles (○) represent low postnatal vitamin C. At P10 and P27 there is a significant difference between prenatal diet group by two-way ANOVA, and by Student’s t-test at P70. N = 7–10 for each group.

Hippocampal volumes were adjusted for total brain weight to identify that the observed effect of prenatal deficiency was specific to the hippocampus and not due to a general retardation of brain growth. Despite this adjustment, hippocampal volumes were consistently lower in prenatally deficient compared to non-deficient counterparts at all time points (P<0.05 or less) (Figure 2).

**Figure 3:**
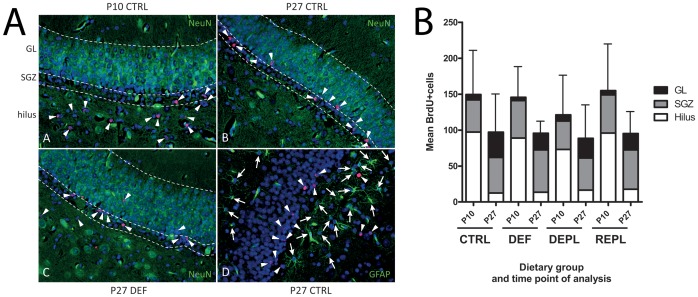
Evaluation of proliferation, survival and migration of precursor cells in the dentate gyrus. A: Representative dentate gyrus area of immunofluorescence-labeled sections used for assessing the location and phenotype of cells born between P5 and P9. Sections of animals sacrificed at either P10 or P27 were analyzed for BrdU-positive cells (red; arrowheads) together with the mature neuronal marker NeuN (Panels A to C) or the astrocyte marker GFAP (D; arrows) (both in green). DAPI (blue) was used to stain cell nuclei. Hatched lines denominate the border zones between hilus, SGZ and GL. **B:** Graphical data from evaluation of immunofluorescence images, example shown in Figure 2b. Results are presented as the mean number of BrdU-positive cells in the different areas of the dentate gyrus for 4–5 regions per animal ± SD for total cell number. N = 7–10 animals per treatment group. We found no difference in the total number of BrdU-positive cells between the different treatment groups analyzed at either P10 or P27 by One-way ANOVA.

To investigate altered proliferation and/or survival of newborn cells in the DG, animals were labeled with 5-bromodeoxyuridine (BrdU) between P5 and P9, and sacrificed at either P10 or P27. Sections were stained for BrdU and NeuN (marker for mature neurons) or GFAP (marker for astrocytes) and the number of BrdU-positive cells counted for each treatment group (n = 7–10) (Figure 3). BrdU-positive cells were identified based on the co-localization of BrdU with DAPI. The DG perimeter was defined as described previously [30]. No significant differences in the mean number of BrdU-positive cells in the DG were found between the different dietary groups at either P10 or P27 (Figure 3), although there was a tendency towards a reduced number of BrdU positive cells at P10 in DEPL (P = 0.22 compared to CTRL; Mann Whitney).

**Figure 4:**
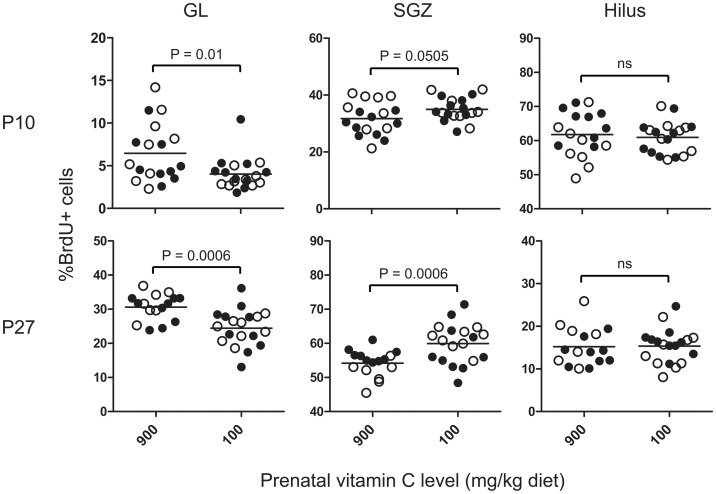
Prenatal vitamin C deficiency causes sustained reduction in postnatal migration of newborn cells into the granule layer. Evaluation of migration pattern of newborn cells according to their location in the different parts of the dentate gyrus at P10 and P27. Results are presented as the percentage of BrdU-positive cells counted in each region. Prenatally-deficient animals were compared to prenatally-sufficient animals by Two-way ANOVA. Closed circles (•) represent high and open circles (○) represent low postnatal vitamin C. N = 7–11 per treatment group. Significantly different mean values are indicated by their respective P-values in the graph; ns indicates no significant difference.

To establish an association between VitC deficiency and impaired migration of newly born cells, the percentage of BrdU positive cells in each sub-region of the DG, i.e., hilus, subgranular zone (SGZ) and granular layer (GL), was calculated. In agreement with previous findings [20], the majority of BrdU-positive cells at P10 were found in the hilus and in the SGZ at P27 (Figure 3). Two-way ANOVA was performed to compare prenatally deficient animals to counterparts receiving high VitC during prenatal development (Figure 4). At both time points (P10 and P27), prenatally-deficient (DEF and REPL) animals had a significantly reduced percentage of BrdU positive cells in the GL (P10: P<0.01 and P27: P<0.001). This was reflected by a significantly higher number of BrdU-positive cells in the SGZ at P27 in prenatally-deficient animals (P<0.001). No differences in the percentage of BrdU-positive cells between prenatally-deficient and prenatally-sufficient animals were observed in the hilus at either time point. These results indicate that prenatal VitC deficiency negatively affects the migration of newborn cells into the GL.

In contrast to a previous study in guinea pigs that used a similar labeling scheme [20], we found that none of the BrdU-positive cells at P27 were positive for NeuN or GFAP (Figure 3), indicating that the newborn cells have not yet matured enough to express either of these markers. However, some of the BrdU-positive cells were positive for doublecortin, a marker for immature neuroblasts (not shown).

### Performance in the Morris Water Maze Test

#### Cued morris water maze test

No statistically significant differences were found between the two groups in any of the analyzed parameters (time to platform localization, total swim distance and cumulative distance to platform).

#### Non-cued morris water maze test

Analysis on quantitative data obtained from the non-cued MWM revealed no significant difference between the three dietary groups at either P24 or P52 tests during the five-day acquisition phase; animals were at the same initial level and equally improved their performance during this learning phase. On the P24 and P52 retention trials (time in platform quadrant, time to first hit on platform area, average distance to platform position and total number of crossings of platform area) we did not find any statistically significant differences between groups, besides that at the P52 test REPL animals displayed a decrease in average swim velocity (cm/sec) significantly different to DEF animals (P<0.05, one-way ANOVA) in the retention trial. There was a significant effect of time between P24 and P52 tests, seen as an overall increase in total swim distance (P<0.001), swim velocity (P<0.001) and frequency of platform crossings (P<0.05), and a reduction in the mean distance to platform area (P<0.05). This corresponds well with the general observation that the animals became more able swimmers with age. However, subsequent reviews of swim patterns [1], [22] did not disclose a difference between groups at either tests (P>0.05), the majority in all groups displaying a random swimming pattern.

## Discussion

The major findings of the present study were that prenatal VitC deficiency affects hippocampal development and leads to a significant and persistent reduction in postnatal hippocampal volume, irrespective of a VitC repletion right after birth. The hippocampal atrophy observed in prenatally vitC-deficient animals may be the consequence of the reduced migration of newborn progenitor cells from the SGZ into the DG.

Our data demonstrates that in spite of selective placental transport of VitC from mother to fetus [36]–[38], the prenatal brain development of the offspring is indeed sensitive to maternal VitC deficiency. Moreover, our findings indicate that VitC deficiency during second and third trimester may trigger adaptive mechanisms, e.g. by up-regulating expression of the vitamin C transporter SVCT2 protein or other protective mechanisms, to increase postnatal VitC uptake and retention in the brain ensuring a rapid repletion once an adequate vitC supply is provided.

The differences in hippocampal volume between prenatally deficient and non-deficient counterparts were maintained even when corrected for brain weight, indicating that the observed effects are not related to retarded brain growth. Moreover, prenatal VitC deficiency leads to a specific reduction in postnatal hippocampal volume which could be found throughout the investigated dietary regimen. The reduction of hippocampal volume was not due to a change in the rate of proliferation or survival of newly generated cells. In humans, 10–15% reductions in hippocampal volume–as those found here–are observed during early signs of dementia or long-term ecstasy or cannabis abuse [39]–[41].

While the postnatal production and survival of neurons was not affected by VitC deficiency, the migration and therefore maturation of newborn cells was negatively affected by prenatal deficiency. We found that the number of BrdU positive cells present in the GL of the DG was significantly lower in prenatally deficient animals (DEF and REPL). Although, ASC is known to affect proliferation and maturation of neuronal precursor cells *in vitro* [42], this is the first report that describes a negative impact of VitC deficiency on progenitor cell development *in vivo*. Although not conclusively demonstrated by our data, it is likely that the impaired migration of newborn cells (despite similar production and survival) is responsible for the decreased hippocampal volume in prenatally deficient compared to control animals.

The brain maintains one of the highest concentrations of VitC in the body and is able to favor this organ even during prolonged states of deficiency [2], [18], [34]. One of the earliest clinical signs of pathological VitC deficiency in guinea pig pups is weight loss [2]. The body weight of DEPL animals was significantly decreased compared to that of CTRL (Table 1). This is surprising since one could imagine that being chronically deficient would be more deleterious than acute postnatal deficiency. One reason could be that these animals were weaned earlier than counterparts (P2 versus P5) resulting in an overall decreased weight gain. However, it may also be that DEPL animals are more sensitive to a sudden decrease in VitC compared to animals who have been accustomed to low levels prenatally. Reviewing brain VitC concentrations at P10, it is evident that repletion occurs highly efficiently, REPL animals being no different than the CTRL group throughout the study. Thus, an alternative explanation could be that that animals subjected to chronically low fetal supply of vitC (DEF animals) have adapted to this environment e.g. by up-regulating SVCT1 & 2 and perhaps ASC recycling to spare as much vitC as possible [14]. The SVCT2 protein is responsible for the majority of the VitC uptake into the brain [43] and its expression, at least in some organs including the cerebellum, has been shown to be increased by VitC depletion [44], [45]. Such a protective mechanism may allow REPL and DEF animals to have a very efficient uptake, compared to DEPL counterparts. In contrast, pups exposed to acute deficiency (DEPL animals) do not have these preventive measures readily available and are therefore more susceptible to a sudden decrease in vitC supply, e.g. requiring time to adjust transport and retention mechanisms. An increased sensitivity to deficiency in guinea pigs going from a high in VitC diet to a very low has previously been shown [46], [47]. Albeit this did not translate into a reduction of the hippocampal volume in the present study, we believe that an effect of depletion would be visible at two months of age. This is supported by our previous study where a postnatal depletion from P7 resulted in 30% reduction of hippocampal neuronal number compared to control counterparts [1].

Several studies have reported that a reduced hippocampal volume may be linked to impaired cognitive capacity [48], [49] and other neurologic disorders [50], [51]. We have reported that postnatal VitC depletion for two months is associated with a diminished spatial memory ability in the non-cued MWM and coherent reductions of neurons in the hippocampus DG, CA1 and CA2-3 fields [1]. To investigate a functional effect of prenatal VitC deficiency, the present animals were also submitted to the MWM test. Cued MWM in CTRL and DEF groups displayed equal performances, demonstrating that vitC deficiency did not induce alterations in basic motor or sensory skills [25], [27]. This was supported by the non-cued MWM acquisition trial, confirming that animals were equally competent prior to the retention tests. However, in contrast to previous findings, we found no difference in memory performances between dietary groups in the non-cued MWM on either P24 or P52; the majority of the animals being random swimmers. Regardless of the reason, limited information can be retrieved from non-cued tests when almost none of the animals display a spatially persistent swim pattern, as this suggests that the potential window of effect was bypassed in the present study. Moreover, we speculate that the difference in hippocampal volume observed here may just not have been large enough to detect an effect by MWM, had we been able to measure it.

Interestingly, we found an overall effect of MWM on ASC homeostasis. Plasma concentrations of ASC in animals subjected to water maze test were significantly lower than those of their non-mazed counterparts (P<0.05). This is likely to be a transient effect due to a reduced feed intake during testing. However, while having comparable ASC levels, the brain displayed a several-fold higher ASC oxidation ratio in mazed compared to non-mazed animals (P<0.001), suggesting that the MWM procedure may impose a degree of stress on the animals leading to enhanced oxidative stress in the brain. This finding is potentially interesting and should be investigated further as increased oxidative stress–if related to the MWM–may impact the interpretation of cognitive performance tests in general.

In conclusion, we show that prenatal VitC deficiency has a persistent negative effect on postnatal hippocampal development as measured by volume reduction over the two month postnatal period monitored in this study. This is most likely due to impaired migration of newborn cells into the GL which may be the consequence of a block in maturation That a marginal deficiency in maternal VitC status leads to a persistent deviation of the hippocampus in offspring supports a pivotal role of VitC during brain development, and emphasizes the requirement for an adequate supply during pregnancy. Marginal vitamin C deficiency is far from uncommon in humans, and may affect large subpopulations including mothers-to-be and young children [52]. This study provides substantial *in vivo* experimental data on a yet unrecognized consequence of marginal, non-scorbutic VitC deficiency that may prove to have implications for nutritional recommendations to pregnant women.

## References

[1] Tveden-Nyborg P, Johansen LK, Raida Z, Villumsen CK, Larsen JO, et al (2009) Vitamin C deficiency in early postnatal life impairs spatial memory and reduces the number of hippocampal neurons in guinea pigs. Am J Clin Nutr 90: 540–6. 19640959 10.3945/ajcn.2009.27954

[2] Lykkesfeldt J, Trueba GP, Poulsen HE, Christen S (2007) Vitamin C deficiency in weanling guinea pigs: differential expression of oxidative stress and DNA repair in liver and brain. Br J Nutr 98: 1116–9. 18309548 10.1017/s0007114507787457

[3] Kratzing CC, Kelly JD, Kratzing JE (1985) Ascorbic acid in fetal rat brain. J Neurochem 44: 1623–4. 3989554 10.1111/j.1471-4159.1985.tb08804.x

[4] Harrison FE, Meredith ME, Dawes SM, Saskowski JL, May JM (2010) Low ascorbic acid and increased oxidative stress in gulo(−/−) mice during development. Brain Res 1349: 143–52. 20599829 10.1016/j.brainres.2010.06.037 PMC2914834

[5] Haramoto M, Tatemoto H, Muto N (2008) Essential role of ascorbic acid in neural differentiation and development: High levels of ascorbic acid 2-glucoside effectively enhance nerve growth factor-induced neurite formation and elongation in PC12 cells. Journal of Health Science 54: 43–9.

[6] Lee JY, Chang MY, Park CH, Kim HY, Kim JH, et al (2003) Ascorbate-induced differentiation of embryonic cortical precursors into neurons and astrocytes. J Neurosci Res 73: 156–65. 12836158 10.1002/jnr.10647

[7] Qiu S, Li L, Weeber EJ, May JM (2007) Ascorbate transport by primary cultured neurons and its role in neuronal function and protection against excitotoxicity. J Neurosci Res 85: 1046–56. 17304569 10.1002/jnr.21204

[8] Harrison FE, Dawes SM, Meredith ME, Babaev VR, Li L, et al (2010) Low vitamin C and increased oxidative stress and cell death in mice that lack the sodium-dependent vitamin C transporter SVCT2. Free Radic Biol Med 49: 821–9. 20541602 10.1016/j.freeradbiomed.2010.06.008 PMC2916678

[9] Sotiriou S, Gispert S, Cheng J, Wang YH, Chen A, et al (2002) Ascorbic-acid transporter Slc23a1 is essential for vitamin C transport into the brain and for perinatal survival. Nature Medicine 8: 514–7. 10.1038/0502-514 11984597

[10] Milby K, Oke A, Adams RN (1982) Detailed mapping of ascorbate distribution in rat brain. Neurosci Lett 28: 169–74. 7070705 10.1016/0304-3940(82)90147-1

[11] Mefford IN, Oke AF, Adams RN (1981) Regional distribution of ascorbate in human brain. Brain Res 212: 223–6. 7225858 10.1016/0006-8993(81)90056-1

[12] Harrison FE, Green RJ, Dawes SM, May JM (2010) Vitamin C distribution and retention in the mouse brain. Brain Res 1348: 181–6. 20570663 10.1016/j.brainres.2010.05.090 PMC2912448

[13] Mun GH, Kim MJ, Lee JH, Kim HJ, Chung YH, et al (2006) Immunohistochemical study of the distribution of sodium-dependent vitamin C transporters in adult rat brain. J Neurosci Res 83: 919–28. 16477646 10.1002/jnr.20751

[14] Tsukaguchi H, Tokui T, Mackenzie B, Berger UV, Chen XZ, et al (1999) A family of mammalian Na+-dependent L-ascorbic acid transporters. Nature 399: 70–5. 10331392 10.1038/19986

[15] Dobbing J, Sands J (1979) Comparative aspects of the brain growth spurt. Early Hum Dev 3: 79–83. 118862 10.1016/0378-3782(79)90022-7

[16] Dobbing J, Sands J (1970) Growth and Development of Brain and Spinal Cord of Guinea Pig. Brain Research 17: 115-&. 10.1016/0006-8993(70)90311-2 5412929

[17] Blandau RJ, Young WC (1939) The effects of delayed fertilization on the development of the guinea pig ovum. American Journal of Anatomy 64: 303–29.

[18] Lykkesfeldt J, Moos T (2005) Age-dependent change in Vitamin C status: a phenomenon of maturation rather than of ageing. Mech Ageing Dev 126: 892–8. 15992612 10.1016/j.mad.2005.03.010

[19] Tveden-Nyborg P, Hasselholt S, Miyashita N, Moos T, Poulsen HE, et al (2011) Chronic Vitamin C Deficiency does not Accelerate Oxidative Stress in Ageing Brains of Guinea Pigs. Basic Clin Pharmacol Toxicol 110: 524–9. 10.1111/j.1742-7843.2011.00852.x 22212866

[20] Guidi S, Ciani E, Severi S, Contestabile A, Bartesaghi R (2005) Postnatal neurogenesis in the dentate gyrus of the guinea pig. Hippocampus 15: 285–301. 15515010 10.1002/hipo.20050

[21] Lykkesfeldt J (2012) Ascorbate and dehydroascorbic acid as biomarkers of oxidative stress: validity of clinical data depends on vacutainer system used. Nutr Res 32: 66–9. 22260866 10.1016/j.nutres.2011.11.005

[22] Dalm S, Grootendorst J, de Kloet ER, Oitzl MS (2000) Quantification of swim patterns in the Morris water maze. Behav Res Methods Instrum Comput 32: 134–9. 10758671 10.3758/bf03200795

[23] Dringenberg HC, Richardson DP, Brien JF, Reynolds JN (2001) Spatial learning in the guinea pig: cued versus non-cued learning, sex differences, and comparison with rats. Behavioural Brain Research 124: 97–101. 11423170 10.1016/s0166-4328(01)00188-7

[24] Vorhees CV, Williams MT (2006) Morris water maze: procedures for assessing spatial and related forms of learning and memory. Nat Protoc 1: 848–58. 17406317 10.1038/nprot.2006.116 PMC2895266

[25] Morris RGM (1981) Spatial Localization Does Not Require the Presence of Local Cues. Learning and Motivation 12: 239–60.

[26] Schenk F, Morris RGM (1985) Dissociation Between Components of Spatial Memory in Rats After Recovery from the Effects of Retrohippocampal Lesions. Experimental Brain Research 58: 11–28. 3987843 10.1007/BF00238949

[27] Morris R (1984) Developments of a water-maze procedure for studying spatial learning in the rat. J Neurosci Methods 11: 47–60. 6471907 10.1016/0165-0270(84)90007-4

[28] Lykkesfeldt J (2000) Determination of ascorbic acid and dehydroascorbic acid in biological samples by high-performance liquid chromatography using subtraction methods: reliable reduction with tris[2-carboxyethyl]phosphine hydrochloride. Anal Biochem 282: 89–93. 10860503 10.1006/abio.2000.4592

[29] Lykkesfeldt J (2007) Ascorbate and dehydroascorbic acid as reliable biomarkers of oxidative stress: analytical reproducibility and long-term stability of plasma samples subjected to acidic deproteinization. Cancer Epidemiol Biomarkers Prev 16: 2513–6. 18006947 10.1158/1055-9965.EPI-07-0639

[30] Rapisarda C, Bacchelli B (1977) The brain of the guinea pig in stereotaxic coordinates. Arch Sci Biol (Bologna ) 61: 1–37. 400095

[31] Sury MD, Agarinis C, Widmer HR, Leib SL, Christen S (2008) JNK is activated but does not mediate hippocampal neuronal apoptosis in experimental neonatal pneumococcal meningitis. Neurobiol Dis 32: 142–50. 18703144 10.1016/j.nbd.2008.07.006 PMC2637370

[32] Tang X, Falls DL, Li X, Lane T, Luskin MB (2007) Antigen-retrieval procedure for bromodeoxyuridine immunolabeling with concurrent labeling of nuclear DNA and antigens damaged by HCl pretreatment. J Neurosci 27: 5837–44. 17537952 10.1523/JNEUROSCI.5048-06.2007 PMC6672250

[33] Lykkesfeldt J (2002) Increased oxidative damage in vitamin C deficiency is accompanied by induction of ascorbic acid recycling capacity in young but not mature guinea pigs. Free Radic Res 36: 567–74. 12150544 10.1080/1071576022411256

[34] Frikke-Schmidt H, Tveden-Nyborg P, Birck MM, Lykkesfeldt J (2011) High dietary fat and cholesterol exacerbates chronic vitamin C deficiency in guinea pigs. Br J Nutr 105: 54–61. 20875184 10.1017/S0007114510003077

[35] Rakipovski G, Raun K, Lykkesfeldt J (2011) Fluctuating hyperglycaemia increases oxidative stress response in lean rats compared to sustained hyperglycaemia despite lower glycaemic exposure. Diab Vasc Dis Res 8: 295–8. 21933843 10.1177/1479164111421033

[36] Norkus EP, Bassi J, Rosso P (1979) Maternal-fetal transfer of ascorbic acid in the guinea pig. J Nutr 109: 2205–12. 512708 10.1093/jn/109.12.2205

[37] Rajan DP, Huang W, Dutta B, Devoe LD, Leibach FH, et al (1999) Human placental sodium-dependent vitamin C transporter (SVCT2): molecular cloning and transport function. Biochem Biophys Res Commun 262: 762–8. 10471399 10.1006/bbrc.1999.1272

[38] Prasad PD, Huang W, Wang H, Leibach FH, Ganapathy V (1998) Transport mechanisms for vitamin C in the JAR human placental choriocarcinoma cell line. Biochim Biophys Acta 1369: 141–51. 9528682 10.1016/s0005-2736(97)00215-0

[39] Wolf H, Grunwald M, Kruggel F, Riedel-Heller SG, Angerhofer S, et al (2001) Hippocampal volume discriminates between normal cognition; questionable and mild dementia in the elderly. Neurobiol Aging 22: 177–86. 11182467 10.1016/s0197-4580(00)00238-4

[40] Yucel M, Solowij N, Respondek C, Whittle S, Fornito A, et al (2008) Regional brain abnormalities associated with long-term heavy cannabis use. Arch Gen Psychiatry 65: 694–701. 18519827 10.1001/archpsyc.65.6.694

[41] Hollander dB, Schouw M, Groot P, Huisman H, Caan M, et al (2012) Preliminary evidence of hippocampal damage in chronic users of ecstasy. J Neurol Neurosurg Psychiatry 83: 83–5. 21444322 10.1136/jnnp.2010.228387

[42] Smith AD, Refsum H (2009) Vitamin B-12 and cognition in the elderly. Am J Clin Nutr 89: 707S–11S. 19116332 10.3945/ajcn.2008.26947D

[43] Harrison FE, May JM (2009) Vitamin C function in the brain: vital role of the ascorbate transporter SVCT2. Free Radic Biol Med 46: 719–30. 19162177 10.1016/j.freeradbiomed.2008.12.018 PMC2649700

[44] Amano A, Aigaki T, Maruyama N, Ishigami A (2010) Ascorbic acid depletion enhances expression of the sodium-dependent vitamin C transporters, SVCT1 and SVCT2, and uptake of ascorbic acid in livers of SMP30/GNL knockout mice. Arch Biochem Biophys 496: 38–44. 20122894 10.1016/j.abb.2010.01.012

[45] Meredith ME, Harrison FE, May JM (2011) Differential regulation of the ascorbic acid transporter SVCT2 during development and in response to ascorbic acid depletion. Biochem Biophys Res Commun 414: 737–42. 22001929 10.1016/j.bbrc.2011.09.146 PMC3210393

[46] Norkus EP, Rosso P (1981) Effects of maternal intake of ascorbic acid on the postnatal metabolism of this vitamin in the guinea pig. J Nutr 111: 624–30. 7218035 10.1093/jn/111.4.624

[47] Norkus EP, Rosso P (1975) Changes in ascorbic acid metabolism of the offspring following high maternal intake of this vitamin in the pregnant guinea pig. Ann N Y Acad Sci 258: 401–9. 1060409 10.1111/j.1749-6632.1975.tb29298.x

[48] Isaacs EB, Lucas A, Chong WK, Wood SJ, Johnson CL, et al (2000) Hippocampal volume and everyday memory in children of very low birth weight. Pediatr Res 47: 713–20. 10832727 10.1203/00006450-200006000-00006

[49] Thompson DK, Wood SJ, Doyle LW, Warfield SK, Lodygensky GA, et al (2008) Neonate hippocampal volumes: prematurity, perinatal predictors, and 2-year outcome. Ann Neurol 63: 642–51. 18384167 10.1002/ana.21367

[50] Mallard EC, Rehn A, Rees S, Tolcos M, Copolov D (1999) Ventriculomegaly and reduced hippocampal volume following intrauterine growth-restriction: implications for the aetiology of schizophrenia. Schizophr Res 40: 11–21. 10541002 10.1016/s0920-9964(99)00041-9

[51] Sapolsky RM (2000) Glucocorticoids and hippocampal atrophy in neuropsychiatric disorders. Arch Gen Psychiatry 57: 925–35. 11015810 10.1001/archpsyc.57.10.925

[52] Tveden-Nyborg P, Lykkesfeldt J (2009) Does vitamin C deficiency result in impaired brain development in infants? Redox Rep 14: 2–6. 19161672 10.1179/135100009X392412

